# Complete Genome Sequence of Streptomyces lunaelactis MM109^T^, Isolated from Cave Moonmilk Deposits

**DOI:** 10.1128/genomeA.00435-18

**Published:** 2018-05-24

**Authors:** Aymeric Naômé, Marta Maciejewska, Magdalena Calusinska, Loïc Martinet, Sinaeda Anderssen, Delphine Adam, Elodie Tenconi, Benoit Deflandre, Wouter Coppieters, Latifa Karim, Marc Hanikenne, Denis Baurain, Philippe Delfosse, Gilles P. van Wezel, Sébastien Rigali

**Affiliations:** a InBioS–Centre for Protein Engineering, Institut de Chimie, University of Liège, Liège, Belgium; b Environmental Research and Innovation Department, Luxembourg Institute of Science and Technology, Esch-sur-Alzette, Luxembourg; c Genomics Platform, GIGA, University of Liège, Liège, Belgium; d InBioS–PhytoSYSTEMS, Functional Genomics and Plant Molecular Imaging, University of Liège, Liège, Belgium; e InBioS–PhytoSYSTEMS, Eukaryotic Phylogenomics, University of Liège, Liège, Belgium; f Molecular Biotechnology, Institute of Biology, Leiden University, Leiden, The Netherlands

## Abstract

Streptomyces lunaelactis MM109^T^ is a ferroverdin A (anticholesterol) producer isolated from cave moonmilk deposits. The complete genome sequence of MM109^T^ was obtained by combining Oxford Nanopore MinION and Illumina HiSeq and MiSeq technologies, revealing an 8.4-Mb linear chromosome and two plasmids, pSLUN1 (127,264 bp, linear) and pSLUN2 (46,827 bp, circular).

## GENOME ANNOUNCEMENT

Streptomyces lunaelactis MM109 is the first Streptomyces type strain originating from cave carbonate speleothems called moonmilk deposits (1). It was isolated in the cave Grotte des Collemboles (Comblain-au-Pont, Belgium) and produces the green-pigmented intracellular iron chelator ferroverdin A (1, 2). S. lunaelactis strains have been found in all analyzed moonmilk deposits in the studied cave (1, 3, 4), suggesting that MM109^T^ could serve as a representative species for investigating the adaptation of streptomycetes to highly oligotrophic and mineral environments. Obtaining the complete genome sequence of MM109^T^ is therefore required to (i) compare the genomic features between cave-dwelling and soil-dwelling streptomycetes, (ii) accurately mine its predisposition at participating in moonmilk genesis (5), and (iii) evaluate its potential at producing natural compounds and identify the ferroverdin A biosynthetic gene cluster.

The complete genome sequence of S. lunaelactis MM109^T^ was obtained by combining sequencing data from three different technologies/approaches, namely, one-dimension Oxford Nanopore MinION (Oxford Nanopore Technologies, UK) and HiSeq and MiSeq (Illumina, CA, USA). The genome was assembled in four steps. First, the Nanopore-generated reads were error corrected, trimmed, and assembled using the long-read assembler Canu version 1.5 (6), which produced three contigs summing to a total length of 8,439,008 bp. Second, the Illumina HiSeq and MiSeq reads were assembled using SPAdes version 3.10.1 (7) and polished in two rounds with the Pilon version 1.22 tool (8), producing 198 contigs with a total length of 8,554,609 bp (contigs ≥ 1 kbp). The polishing step resolved inconsistencies between the Illumina draft assembly and Illumina read mapping in an iterative fashion until self-consistency. The third step consisted of improving the initial Nanopore assembly in eight successive rounds of polishing with the Illumina reads using Pilon, bringing the total length to 8,589,330 bp with 7,827 coding sequences. Finally (step 4), the genome was manually curated to resolve residual misassemblies, indels, and base differences observed upon the mapping of the Illumina assembly contigs (from step 2) onto the polished Nanopore assembly (from step 3). Inspection of the average Illumina sequencing coverage of the three assembled contigs revealed that the two shorter contigs were likely to be low-copy-number plasmids (named pSLUN1 and pSLUN2), with coverages (541× and 608×x) three-fold greater than that of the chromosome (177×). The presence of repeated sequences at both ends of pSLUN2 suggested a circular plasmid, which was circularized using the Circlator software (9). The automatic annotation of the genome was performed with the NCBI Prokaryotic Genome Annotation Pipeline (PGAP) upon submission to GenBank.

The final assembly of the S. lunaelactis MM109^T^ genome revealed a single linear chromosome with a size of 8,396,100 bp, and two plasmids, the linear pSLUN1 (127,264 bp) and the circular pSLUN2 (46,827 bp). The genome displayed an overall G+C content of 69.75%. A total of 7,718 protein-coding genes were predicted, as well as 66 tRNAs and 6 rRNA operons. Genome mining allowed the identification from nucleotide positions 4637038 to 4656557 of a 17-membered biosynthetic cluster presenting 85% and 91% amino acid identity and similarity, respectively, with the *fev* cluster (GenBank accession number AB689797) of another ferroverdin A producer, Streptomyces sp. strain WK53-44.

### Accession number(s).

This whole-genome shotgun project has been deposited in DDBJ/ENA/GenBank under the accession numbers CP026304 for the chromosome and CP026305 and CP026306 for the plasmids pSLUN1 and pSLUN2, respectively. Raw sequence data are also available from the NCBI Sequence Read Archive under accession numbers SRR6475285 to SRR6475287.
